# Supplementary figures and images for: Pip5k1γ regulates axon formation by limiting Rap1 activity
Source: Life Sci Alliance. 2024 Mar 4;7(5):e202302383. doi: 10.26508/lsa.202302383 (PMC10912816; doi:10.26508/lsa.202302383)

Figure S2

A

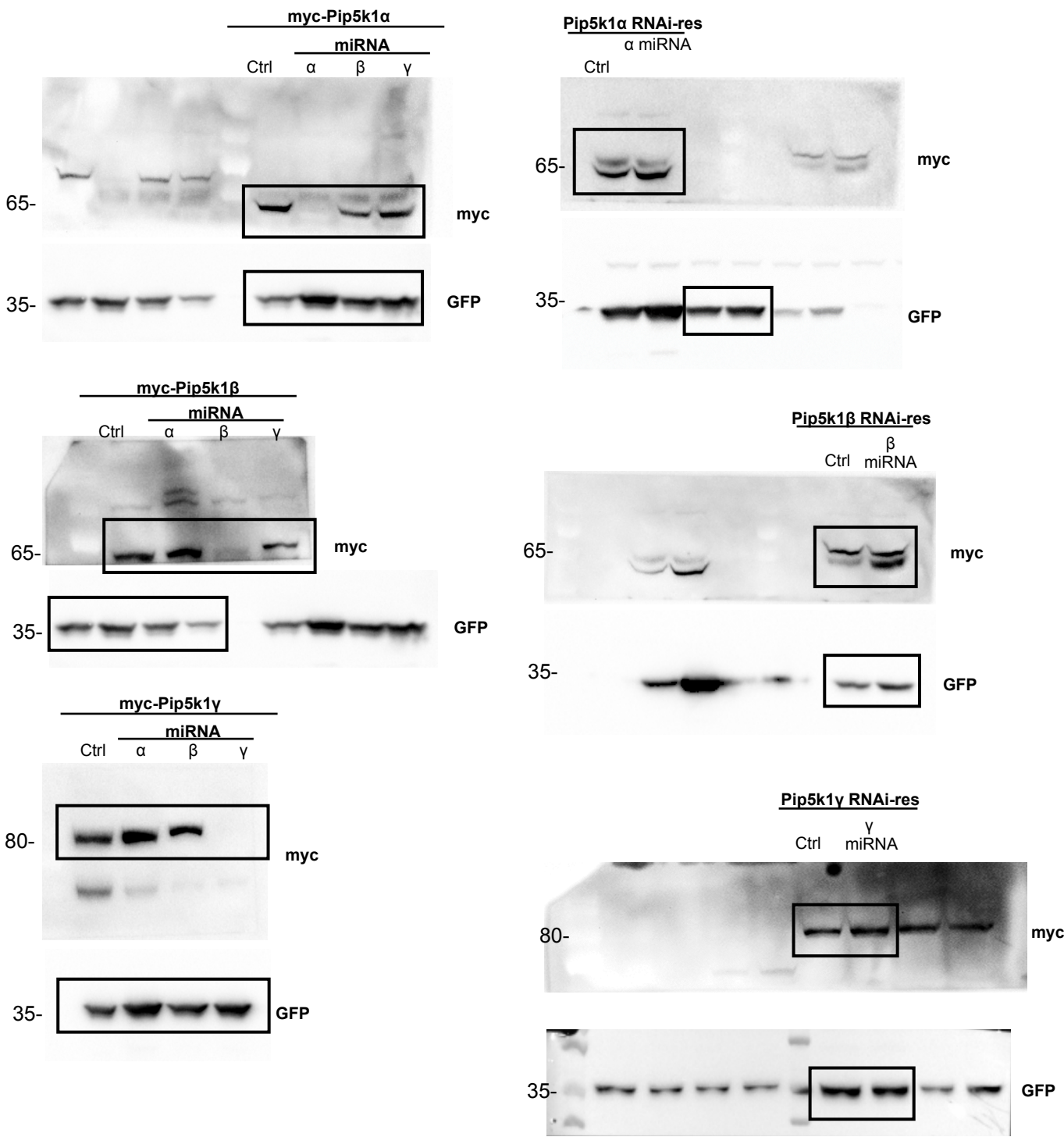

Supplement: Supplementary file 1 [file LSA-2023-02383_SdataFS2.pdf]

Figure S3

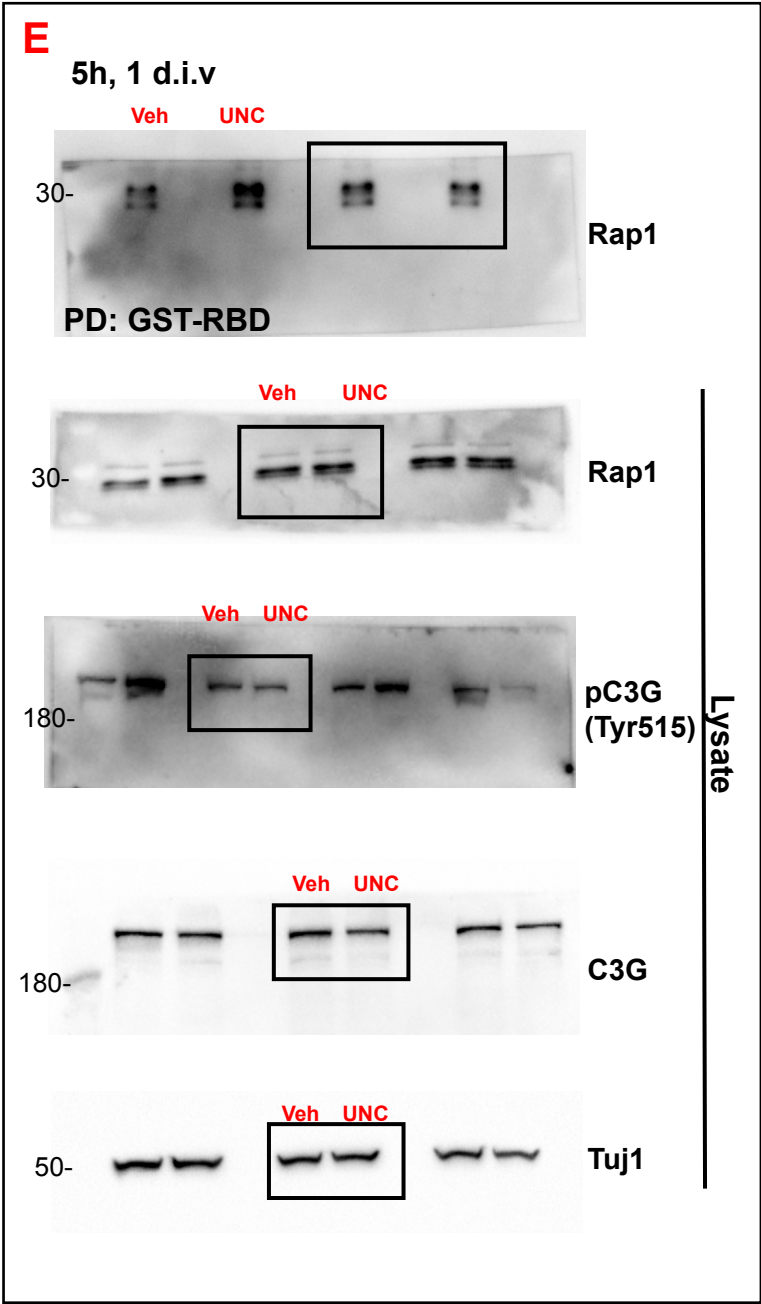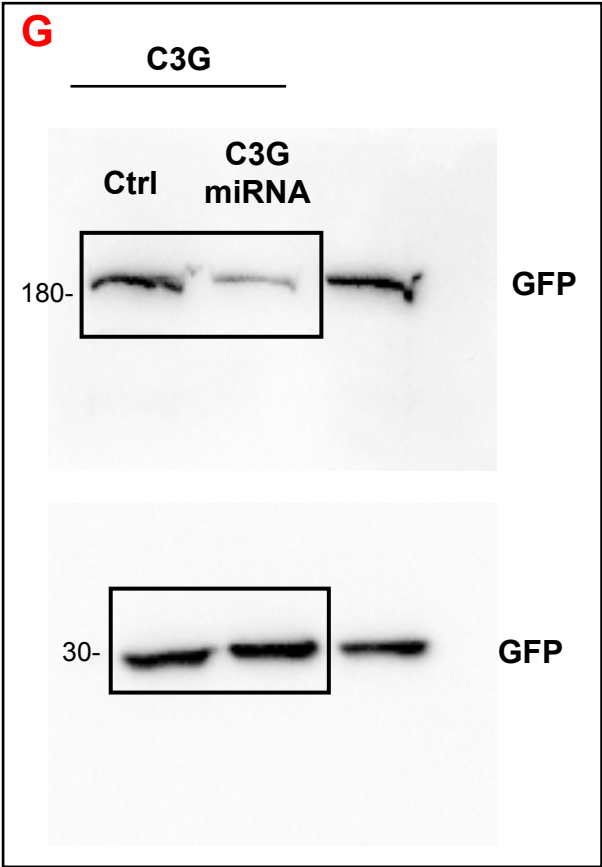

Supplement: Supplementary file 2 [file LSA-2023-02383_SdataFS3.pdf]

Figure 3

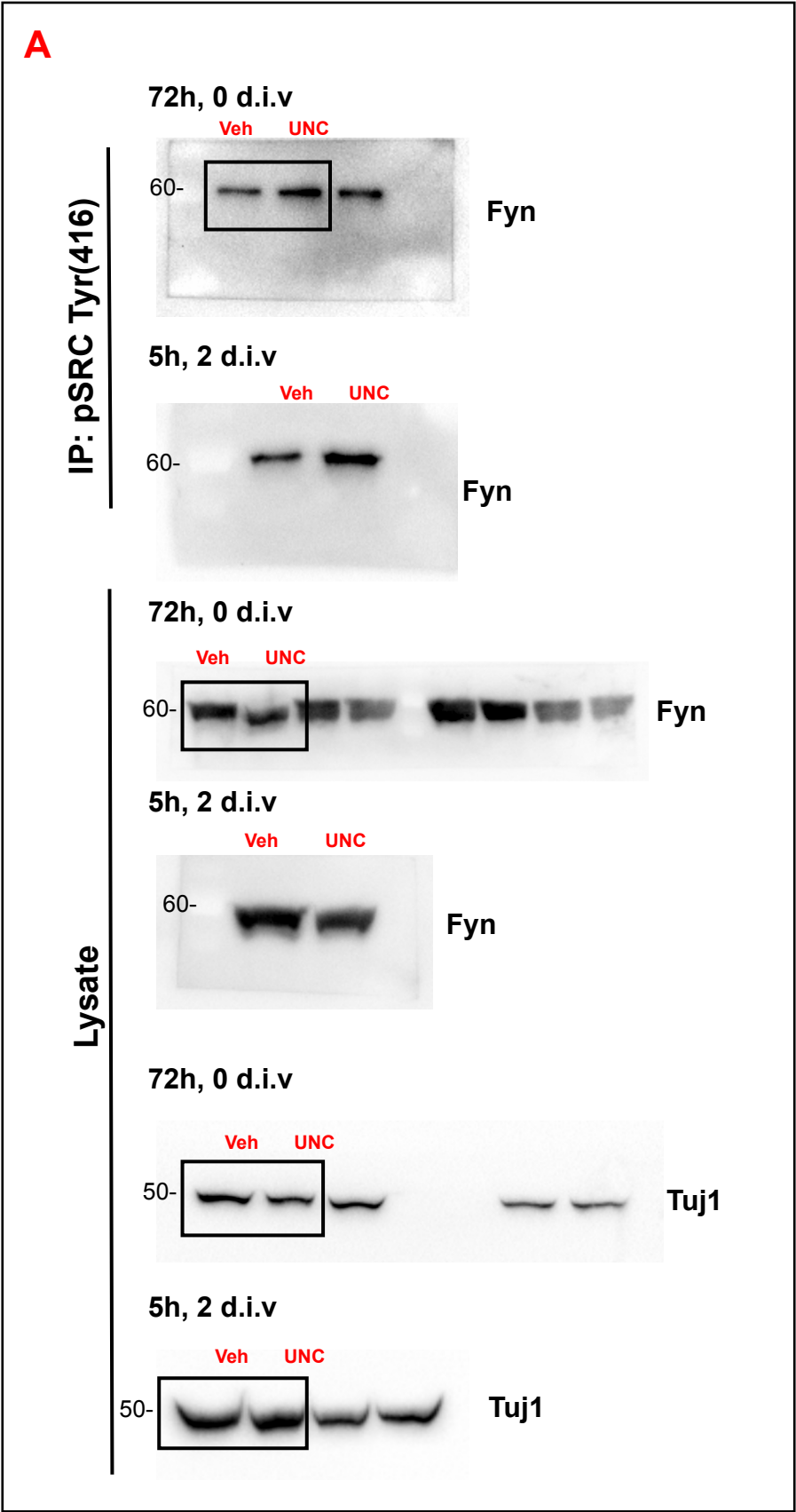

Figure 3

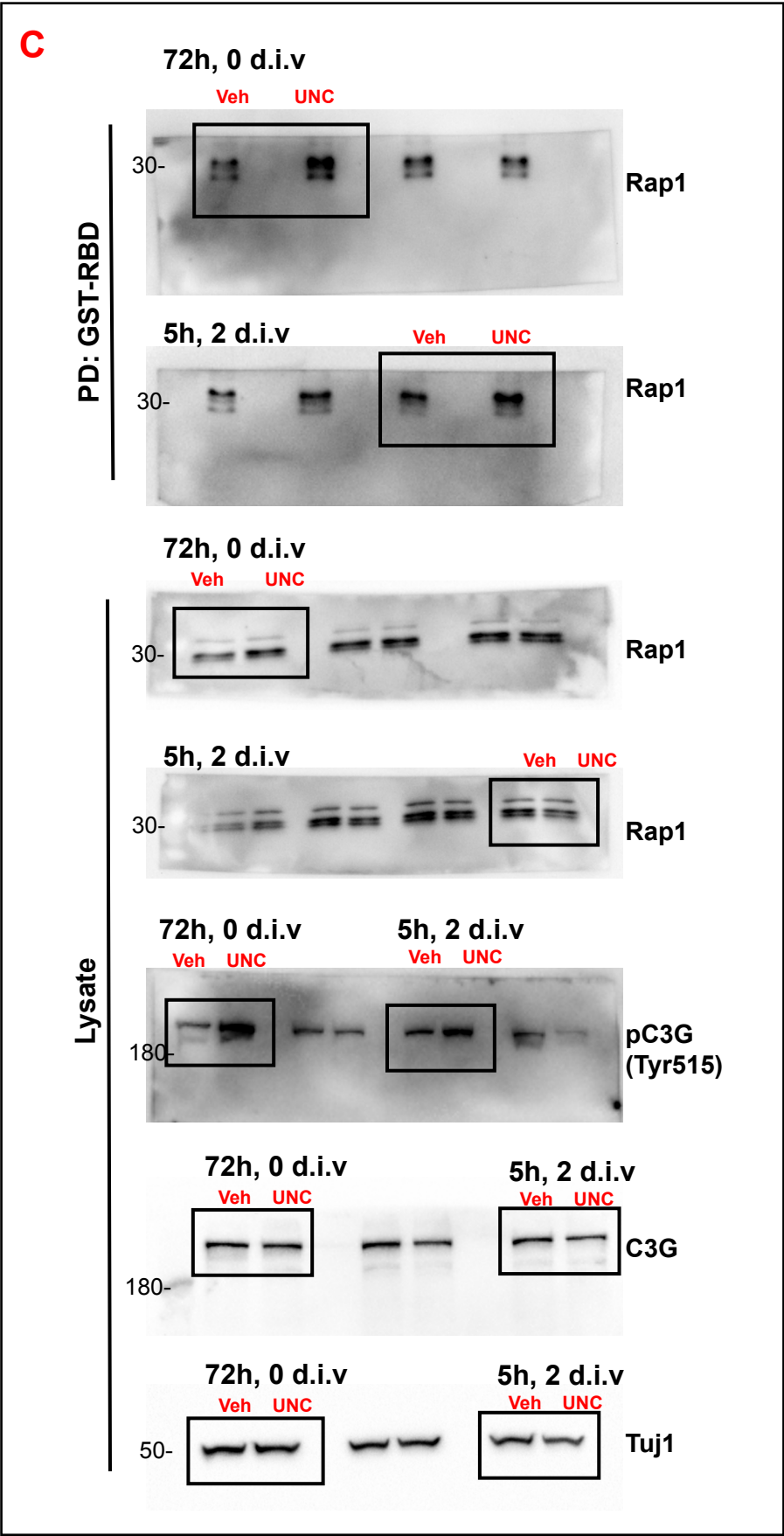

Supplement: Supplementary file 3 [file LSA-2023-02383_SdataF3.pdf]

Figure 6

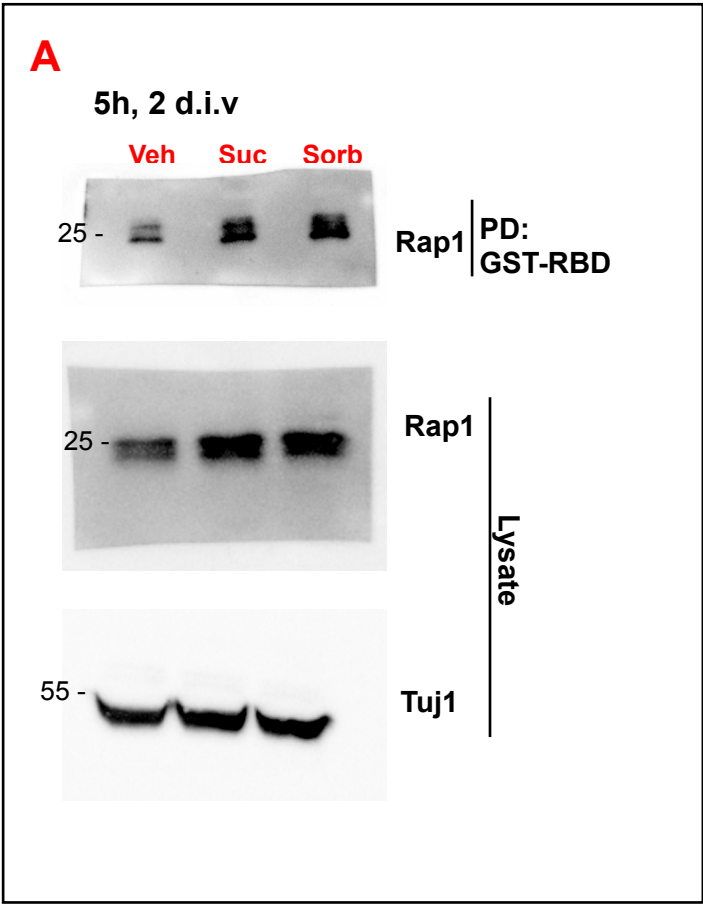

Supplement: Supplementary file 4 [file LSA-2023-02383_SdataF6.pdf]
